# Downregulation of LINC02615 Is Correlated with The Breast Cancer Progress: A Novel Biomarker for Differential Identification of Breast Cancer Tissues 

**DOI:** 10.22074/cellj.2021.7283

**Published:** 2021-08-29

**Authors:** Sayed Rasoul Zaker, Kamran Ghaedi

**Affiliations:** 1 Department of Plant and Animal Biology, Faculty of Biological Science and Technology, University of Isfahan, Isfahan, Iran; 2 Department of Cellular Biotechnology, Cell Science Research Center, Royan Institute for Biotechnology, ACECR, Isfahan, Iran; 3 Department of Cell and Molecular Biology and Microbiology, Faculty of Biological Science and Technology, University of Isfahan, Iran

**Keywords:** Biomarker, Breast Cancer, LINC02615, LincRNA, Obesity

## Abstract

**Objective:**

Breast cancer is one of the most frequent types of cancer with a gradually increasing incidence in developing countries. The aim of this study was to assess modulation of LINC02615 levels in breast cancer progress, using pairwise breast cancer and healthy control tissue samples with regard to the obesity and other conditions, as estrogen receptor (ER) expression.

**Materials and Methods:**

In this cohort study, the genes, microRNAs (miRNAs) and long non-coding RNAs (lncRNAs) in several important pathways of chromosomal instability, apoptosis and proliferation were analyzed through in silico studies pinpointing the important genes which were responsible for the breast cancer incidence. Then, the respective miRNAs and lncRNAs were selected by relevant databases. At the next step, Lncbase was used for interaction analysis of selected miRNAs and LncRNAs, which resulted in final selection of LINC02615. Total RNA was isolated from 24 pairwise breast cancer and healthy control tissue samples. Expression profile of LINC02615 was assessed using quantitative reverse transcription polymerase chain reaction (qRT-PCR). Correlation between LINC02615 expression and clinicopathological characteristics were analyzed using Pearson’s Chi-square test in breast cancer patients.

**Results:**

Data demonstrated that expression of LINC02615 was significantly downregulated in breast cancer tissues compared to the healthy controls (P=0.046). In particular, the relative LINC02615 expression was significantly different in breast cancer tissues especially in obese patients compared to those persons without obesity (P=0.047). Furthermore, a significant difference in LINC02615 level was found between the high and low ER expressions (P=0.014). However, the aberrant expression of LINC02615 was significantly related to physical activity and diabetes disease as well as the stress and age at menopause (P=0.028, P=0.046, P=0.047 and P=0.025, respectively).

**Conclusion:**

Taken together, we suggest that LINC02615 downregulation may be related to the risk of breast cancer in Iranian patients. Thus, it may serve as a novel biomarker for identification of breast cancer tissues.

## Introduction

Breast cancer is one of the most frequent invasive malignancies, as the main cause of cancer-related death among females worldwide (1-3). In Iranian population, breast cancer is the most common type of cancer with a peak of incidence among females occurring mostly in the fourth and fifth decades of life (3, 4). Unfortunately, a gradually increasing incidence *in* breast cancer has been reported in Iranian women at age 15-79 years during recent 30 years, seriously threatening the life and health (5). Classification of breast cancer into the different subtypes is performed according to the tumor size, metastasis to lymph nodes or other parts, expression of human epidermal growth factor receptor 2 (HER2), estrogen receptors (ER) and progesterone receptors (PR) (6). 

Long noncoding RNAs (lncRNAs), are a family of gene transcripts which play a significant regulatory role in a variety of cellular and biological processes. Abnormal expressions of lncRNAs are reported to significantly contribute to cancer incidence and progression, especially in breast cancer (7-9). Since lncRNAs exert a critical role in breast cancer etiology and progression, they may have potential diagnostic and prognostic biomarker capability for different malignancies (10). lncRNAs are identified to regulate gene expression and chromatin structure while they have exhibited different expression patterns in various cancers. These are associated with cancer progression (11). Therefore, they can be used as cancer diagnostic and/or prognostic biomarkers and targeted therapeutic approach. Although, a large number of lncRNAs have been annotated across human cancer tissues using high-throughput sequencing technologies, only a portion of them have still been validated (12). lncRNA LINC02615 (long intergenic non-protein coding RNA 2615) locates on the long arm of chromosome 4 (4q28.2) and its transcript length is 742 bases. The functional role of this lncRNA and its expression in breast cancer remains to be uncovered. 

The aim of this study was to identify the relevance of LINC02615 to pathogenesis of breast cancer and assessment of co-expression of this lncRNA in cancer progress with regard to the obesity and other conditions as ER, PR and HER expressions. 

Therefore, to address whether LINC02615 modulation is correlated with the breast cancer progress and incidence, we assessed expression of LINC02615 in 24 pairwise cancer and adjacent control tissues using quantitative reverse transcription polymerase chain reaction (qRT-PCR). Our results revealed that LINC02615 may be used as a novel diagnostic and prognostic biomarker in breast cancer.

## Materials and Methods

This study was a cohort study to investigate correlation between the incidences of breast cancer and modulation of LINC02615.

### Bioinformatics analysis

At the first stage, four important signaling pathways related to the pathogenesis of breast cancer were selected to analyze, including: i. Chromosomal instability, ii. Apoptosis, iii. Cell cycle progression, and iv. Proliferation survival translation (13-20). Key cancer genes in these pathways were identified through a literature review, WikiPathway (https://wikipathways.org) and KEGG pathway enrichment analysis (https://www.genome.jp). At the next step, the respective miRNAs were selected using online prediction databases as miRDB (www.mirdb.org) (21), miRTarbase (mirtarbase.mbc.nctu.edu.tw) (22), Tarbase version 8 (carolina.imis.athena-innovation.gr) (23) and DIANA-microT (diana.imis.athena-innovation.gr) (24) were utilized to predict relevant miRNAs targeting the selected key genes. Then, functional enrichment analysis MIEAA database (https://bio.tools › miEAA) was used to identify the role of predicted miRNAs in cancer. Selecting lncRNA associated with predicted miRNAs was accomplished using LncBase experimental version 2 software (carolina.imis.athena-innovation.gr › diana_tools › web › index-experimental). Finally, co-expression of these genes and predicted lncRNAs was assessed using Co-LncRNA database (http://www.bio bigdata.com/CoLncRNA/) (25).

### Patients’ samples

All samples were obtained upon signing of the informed consent by the breast cancer patients who were referred to Alzahra hospital (Central hospital of Isfahan University of Medical Sciences), Isfahan, Iran. Twenty-four patients were selected who have been diagnosed by a gynecologist after pathological observation. Twenty-four pairs of matched samples of breast cancer and healthy tissues were isolated by a gynecologist. To validate whether those tissues are cancerous or not, a portion of each sample were sent for pathological observations. The remaining part of each sample was put in RLT buffer (Qiagen, Germany) and kept in -80˚C for further usage.

All protocols and methodology of this work was performed according to the Declaration of Helsinki, reviewed and approved by the Ethical Committee of the University (IR.UI.REC.1398.052).

### RNA extraction and cDNA synthesis

Total cellular RNA was extracted from all tissues using TRIzol reagent (YTA, Iran), according to the manufacturer’s instruction. RNA concentration and integrity were assessed by NanoDrop spectrophotometer (Thermo Fisher Scientific, USA) and 1% agarose gel electrophoresis, respectively. Finally, cDNA synthesis was carried out by cDNA Synthesis Kit (Thermo Fisher Scientific, USA) using oligo (dt) nucleotides.

### Primer design and quantitative reverse transcription polymerase chain reaction analysis

The resulting cDNA products were amplified using YTA SYBR Green qPCR MasterMix 2X (YTA) the specific primers of *GAPDH*, as an internal control, and lncRNA LINC02615 in a Mic qPCR cycler (Table 1). Quantitative reverse transcription polymerase chain reaction (qRT-PCR) was carried out at 95˚C for 10 minutes, followed by 40 cycles using the following protocol: 95˚C for 15 seconds, 60˚C for 15 seconds, and 72˚C for 1 minute. Gene Runner software and OligoCalc tool (biotools. nubic.northwestern.edu) were utilized to design the primers for *GAPDH* and LINC02615. LINC02615 is located on chromosome 4q28.2. Specific primers for LINC02615 were designed to amplify a fragment of the exon 9 (the last exon) with a product size of 177 bp. The specificity of primers were again analyzed using *NCBI Primer BLAST* server (http://blast.ncbi.nlm.nih.gov/Blast. cgi). Relative expression of the LINC02615 transcript was calculated and reported based on ΔΔCt method. Standard curves for the primer efficiency in qRT-PCR are indicated in Figure S1 (See Supplementary Online Information at www.celljournal.org).

### Statistical analysis

SPSS program (version 21.0, IBM Co., USA) was utilized to perform statistical analysis. Initially, the normality of data distribution was examined by the Shapiro-Wilk and Kolmogorov Smirnov normality test (KS-test). The receiver operating characteristic (ROC) curve analysis was used to predict lncRNA expression cutoff point and evaluate the diagnostic specificity and sensitivity of LINC02615 level as a biomarker to differentiate between breast cancer groups and non-cancer individuals. In the present study, correlation of LINC02615 expression with clinicopathological characteristics was analyzed using Pearson’s Chi-square test in breast cancer patients. However, P<0.05 was considered statistically significant. All experiments were performed in duplicate and the results were presented as the mean ± standard deviation (SD) in the current study

## Results

Clinical and pathological characteristic of the patients are summarized in Table 2.

### In silico studies

According to WikiPathway and KEGG pathway enrichment analysis, the key genes were selected in the four pathways, including chromosomal instability, apoptosis, and cell cycle progression, proliferation survival translation. The list of these genes is presented in Table S1 (See Supplementary Online Information at www.celljournal.org).

These genes may tightly be associated with the pathogenesis of breast cancer. Furthermore, Table S2 (See Supplementary Online Information at www.celljournal. org) shows the predicted miRNAs regulating one-third of the selected key genes at the mRNA levels.

**Fig.1:**
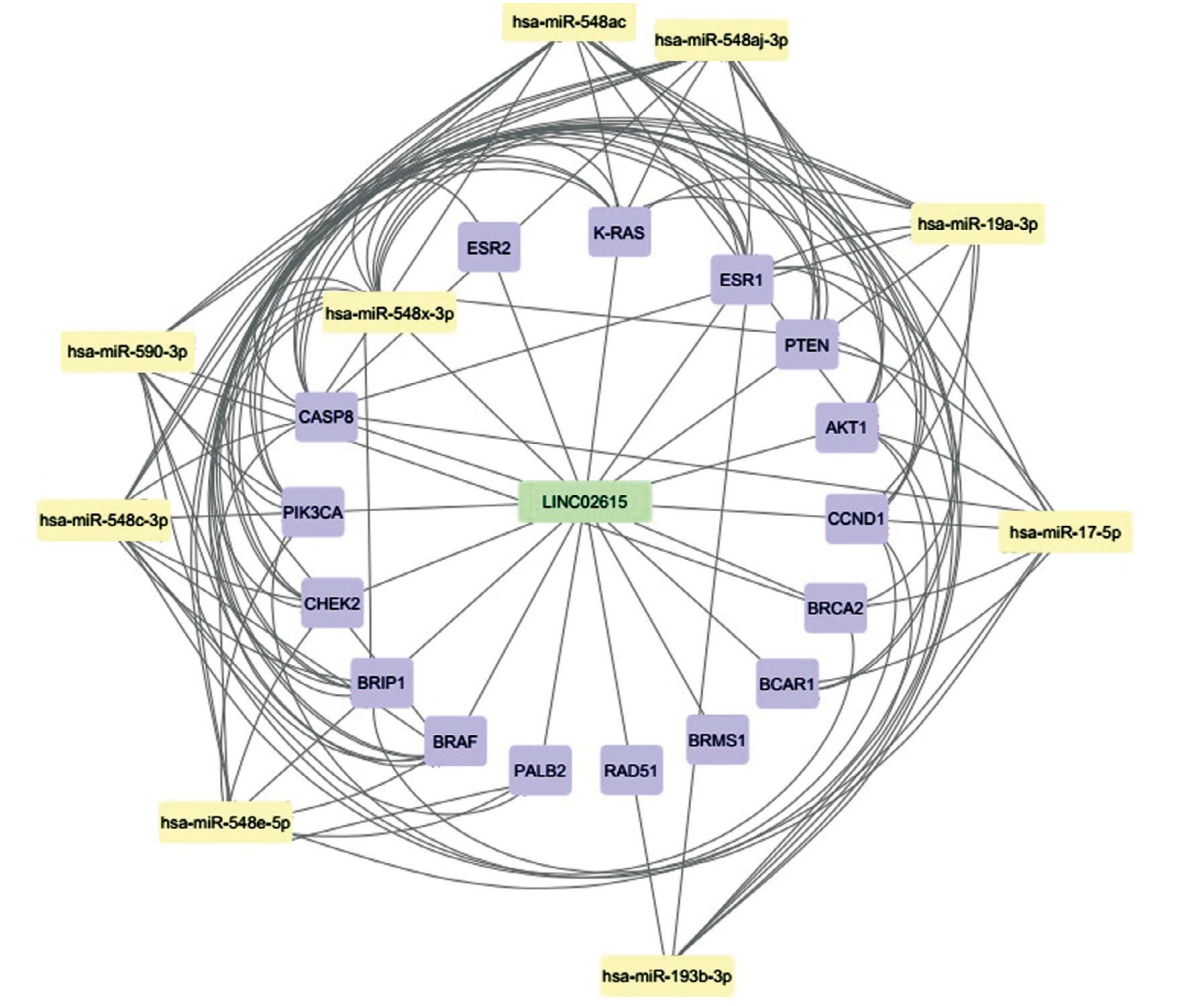
The most important miRNAs associated with LINC02615 and their targets which are visualized in Cytoscape.

In order to identify the most important miRNAs, the predicted miRNAs were shared (Table S3, See Supplementary Online Information at www.celljournal.org, Fig .1).

**Table 1:** Specific primer sequences of LINC02615 and *GAPDH*


Gene	Primer sequence (5′ to 3ˊ)	Tm (˚C)	GC%	Amplicon length (bp)

*LINC02615*	F: GTGATAGATGGAAACCCTTGTTC	60.9	43	177
	R: GATGGGCTGTAACAATGAATGC	60.1	45	
*GAPDH*	F: GCTCTCTGCTCCTCCTGTTC	62.5	60	115
	R: ACGACCAAATCCGTTGACTC	58.4	50	


Tm; Melting temperature.

**Table 2:** Pathophysiological features of participates in the current research


Characteristics		n=24

Age (Y)		
	Mean ± SD	47.56 ± 12.46
HER2 expression		
	Positive	4
	Negative	2
	NA	18
ER expression		
	Positive	3
	Negative	3
	NA	18
PR expression		
	Positive	2
	Negative	4
	NA	18
Tumor stage		
	I/II	8
	III/IV	6
	NA	10
Tumor grade		
	I/II	12
	III	3
	NA	15
Tumor size (cm)		
	5≥	12
	5≤	15
	NA	7
Family history of breast cancer		
	Positive	3
	Negative	3
	NA	18
Physical activity		
	High	4
	Intermediate/low	4
	NA	16
Obesity		
	Yes	11
	No	13
	NA	0
Dietary factors		
1- Fat intake		
	High	6
	Intermediate/low	5
	NA	13
2- Red meat intake		
	High/Intermediate	5
	Low	6
	NA	13
3- Dairy intake		
	High/Intermediate	10
	Low	2
	NA	12
Income		
	High	2
	Intermediate	4
	Low	5
	NA	13
Diabetes disease		
	Yes	2
	No	2
	NA	20
Stress		
	Yes	8
	No	1
	NA	15
Marital status		
	Married	16
	Never married/single	0
	NA	8
Marital age (Y)		
	20≥	13
	20≤	3
	NA	8
Age at first pregnancy (Y)		
	20≥	12
	20≤	3
	NA	9
Breast feeding history		
	Yes	10
	No	2
	Missing data	12
Birth control pills (hormonal)		
	Yes	8
	No	3
	NA	13
Age at menarche (Y)		
	15≥	14
	15≤	0
	NA	10
Age at menopause (Y)		
	47≥	2
	47≤	3
	NA	19


ER; Estrogen receptor, PR; Progesterone receptor, HER2; Human epithelial growth factor receptor 2, and NA; Not applicable.

**Fig.2:**
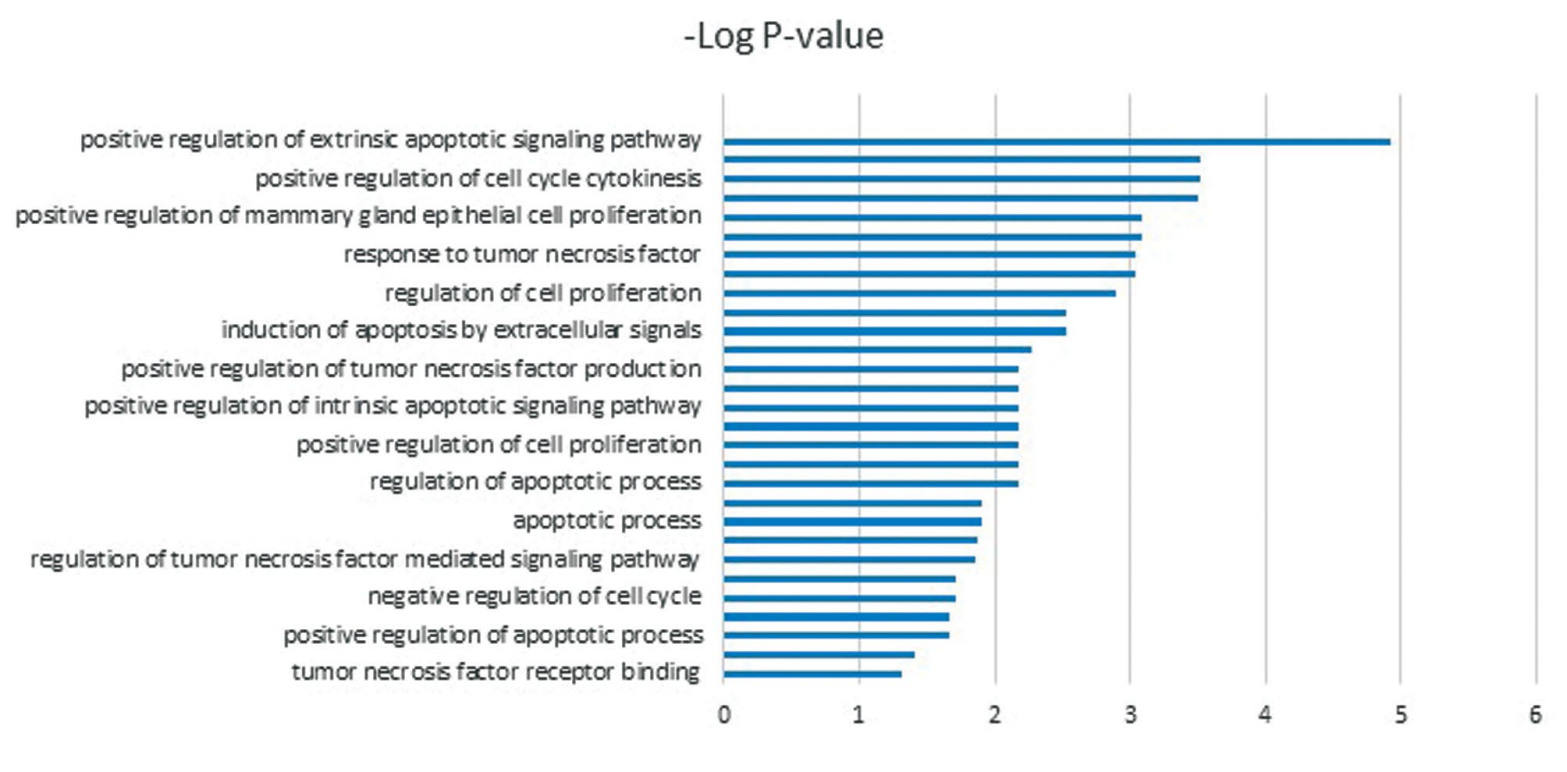
The most important function of predicted miRNAs in cancer.

As shown in Table S4 (See Supplementary Online Information at www.celljournal.org) and Figure 2, functional analysis demonstrated these miRNAs have an essential role in cancer initiation and progression, including regulation of chromosomal instability, apoptosis, cell cycle progression and proliferation survival translation.

Negative/positive co-expressions were found between LINC02615 and the selected key genes using Co-LncRNA database (Table S5, See Supplementary Online Information at www.celljournal.org). Therefore, LINC02615 was considered for further analysis. Finally, the bioinformatics analysis revealed that eight miRNAs are probably associated with LINC02615, including hsa-miR-548aj-5p, hsa-miR-153-5p, hsa-miR-548x-5p, hsa-miR-5590-3p, hsa-miR-142-5p, hsa-miR-548g-5p, hsa-miR-129-5p and hsa-miR-4753- 3p (Fig .1). Furthermore, hsa-miR-129-5p was found as a miRNA with the highest score, 0.873, amongst the aforementioned miRNAs.

### Comparing the expression profile of LINC02615 among the breast cancer patients and controls

**Fig.3:**
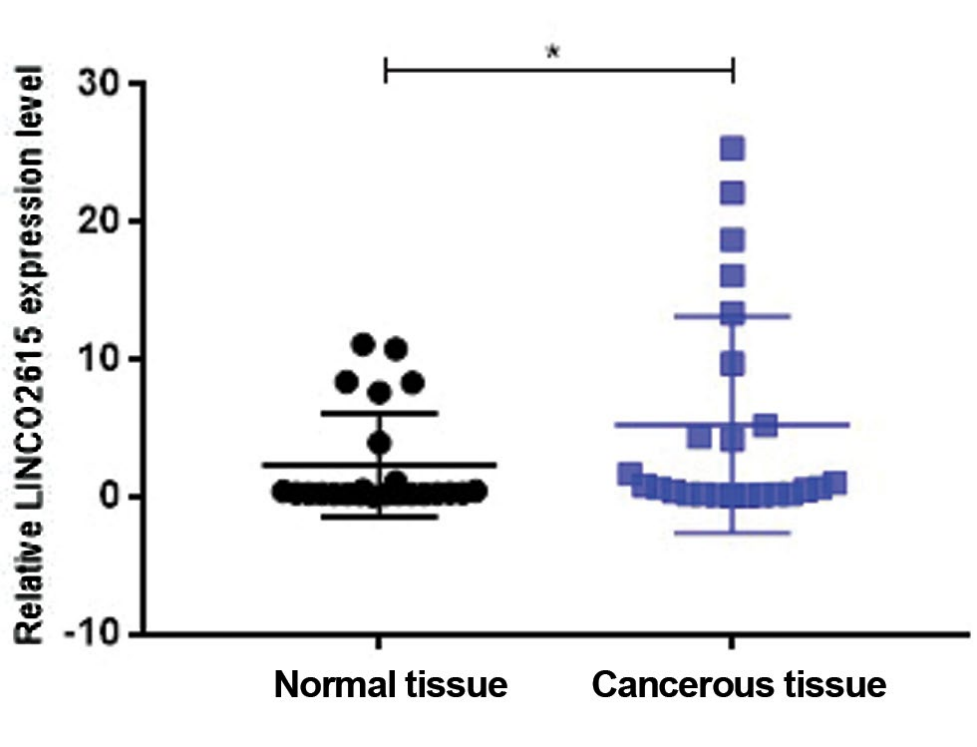
Correlation between expression profile of LINC02615 and cancer incidence. *; Indicates significant difference at P<0.05.

The relative LINC02615 expression level in 24 breast tissues of cancer patients and healthy individuals were assessed by qRT-PCR. Results identified that LINC02615 expression level was significantly lower in cancer tissues compared to the healthy tissues, by P value of 0.043 (Fig .3).

The relative expression levels of LINC02615 in breast cancer tissues compared to the control counterparts. Significant downregulation of LINC02615 expression level in breast cancer tissues compared to the normal samples with P<0.05.

**Fig.4:**
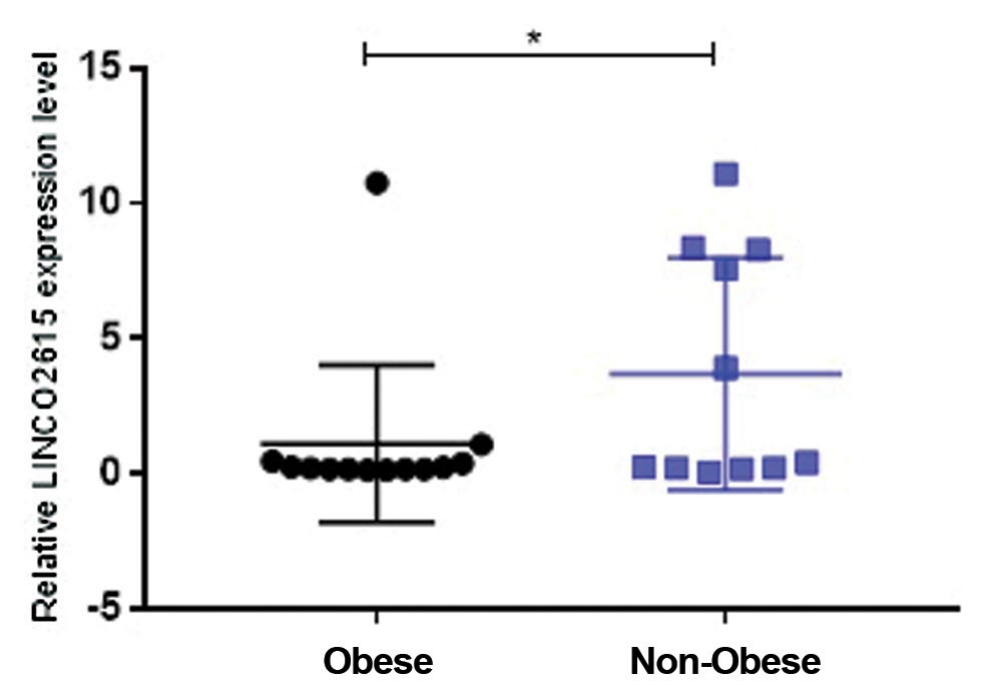
Comparative levels of LINC02615 expression in breast cancer tissue of patients with obesity versus non-obese patients. *; Is significant difference at P<0.05.

Subsequently, correlation of the relative LINC02615 expression level with other clinical features of breast cancer (such as ER, PR, and HER2 expression as well as cancer stage and grade) was assessed. As shown in Table S6 (See Supplementary Online Information at www.celljournal. org) results demonstrated that differential expression of LINC02615 was significantly associated with ER expression and physical activity as well as diabetes disease, stress and age at menopause, in breast cancer patients (P=0.014, P=0.028, P=0.046, P=0.047 and P=0.025, respectively). Furthermore, LINC02615 expression level in breast cancer tissue of patients with obesity was significantly higher than that in those without obesity (P=0.047, Fig .4).

The relative expression levels of LINC02615 in the breast cancer patients with obesity were compared to the patients without obesity. Significant decreased expression level of LINC02615 was detected in the breast cancer patients without obesity compared to the patients with obesity (*P<0.05).

However, differential expression of LINC02615 was not significantly related to HER2 and PR expression as well as stage, grade and size of the tumor (P=0.439, P=0.083, P=0.078, P=0.08 and P=0.252 respectively). Moreover, difference of LINC02615 expression was not significantly associated with family history of breast cancer, income, marital age, age at first pregnancy, breastfeeding history and birth control pills as well as dietary factors including fat, red meat and dairy intakes (P=0.273, P=0.209, P=0.473, P=0.448, P=0.067, P=0.338, P=0.621, P=0.387 and P=0.121, respectively). 

## Discussion

In many low-income countries, besides of the gradually rising incidence of breast cancer, limited healthcare resources along with inadequate strategies for diagnosis lead to face a fundamental health challenge (26, 27). In the current study, the key genes in chromosomal instability, apoptosis, cell cycle progression and proliferation survival translation, involved in the pathogenesis of breast cancer (13-20), were identified using WikiPathway and KEGG pathway enrichment analysis. Since miRNAs have been related to various biological and pathological processes including cancer risk, development, progression, drug resistance and survival (28, 29), a number miRNAs which regulate at least one-third of the selected genes were predicted using online prediction software miRDB, miRTarbase, Tarbase, and DIANA-microT. At the next step, negative/positive co-expressions were illustrated between LINC02615 and the selected key genes using Co-LncRNA database and eventually LINC02615 was considered for further investigations. Results demonstrated that LINC02615 level was significantly decreased in the breast cancer tissues compared to the normal samples. Due to the lack of any publication regarding to LINC02615, we tried to find direct relations of this lncRNA with pathologic conditions through bioinformatics analysis including LncDisease tool (LncDisease.bio.tools). However there was no relevant predicted data in this case. Recently, some of lincRNAs have been reported to be aberrantly expressed in different types of malignancies including breast cancer. For example, it was identified that LINC00152 and LINC01082 were significantly downregulated in tumor tissues, in comparison with their adjacent non-cancer tissues (12). In this regard, Chen et al. (30) demonstrated that LINC00628 expression was significantly decreased in breast cancer patients and cell lines. Low expression of this lincRNA was related to poor prognosis and lower overall survival (OS) rate in patients with breast cancer. Another study reported that increased expression of LINC-NORAD was associated with proliferation, invasion and migration of breast cancer cells and it was related to poor outcome (31). In addition, Vishnubalaji et al. (32) suggested that lnc-LRR1–1, AC015712.5, lnc-SPP2–3, lnc-ODF3B-2, lnc-MAP9-2, and lnc-LAMB3-1 expression are related to better disease-free survival (DFS), while the expression of LINC01614 and LINC01235 were associated with worse DFS. Furthermore, they showed that expression of MIR205HG, lnc-SPP2-3, lnc-MAP2K6-5 and FGF14-AS2 were related to better OS, while the expression of LINC01235 were associated with worse OS. However, they demonstrated that LINC01614 was overexpressed in HER2+, PR+ and ER+ patients. Some studies revealed that linc-ROR can act as an oncogene in breast cancer tissue and it was related to lymph node metastasis and worse prognosis (33-35).

On the other hand, stratified association analysis was performed between LINC02615 expression at the mRNA level and clinical information including hormonal receptor status (ER, PR and HER2), stage and grade of breast cancer, obesity or other factors. Statistical data revealed that LINC02615 dysregulation was significantly related to ER expression and physical activity as well as diabetes disease, stress and age at menopause in the breast cancer patients. Surprisingly, expression of LINC02615 in patients with obesity was significantly higher than those who were not obese. However, aberrant expression of LINC02615 was not significantly correlated with HER2 and PR expressions as well as stage, grade and size of the tumor. In addition, differential expression of LINC02615 was not significantly related to the family history of breast cancer, income, marital age, age at first pregnancy, breast feeding history and birth control pills as well as dietary factors such as fat, red meat and dairy intakes.

## Conclusion

Taken together, our results provide the first report of association between LINC00628 expression and breast cancer. The results suggested that LINC00628 may act as a tumor suppressor in breast cancer tissue and could act as a novel differential diagnostic biomarker in these patients. As the present research is the first report on functional aspect of LINC00628, additional evidences are required to clarify the exact role of LINC00628 in cancer progression and especially breast cancer. Furthermore, since the aberrant expression of this lincRNA was significantly linked to the ER expression, physical activity, diabetes disease, stress, age at menopause and obesity in breast cancer patients, LINC00628 expression can be proposed as a biomarker in patients with breast cancer. However, the validity of our hypothesis should be evaluated in a larger sample size of the patients, in future independent cohort studies. 

## Supplementary PDF


