# Supplementary material for: Downregulation of LINC02615 Is Correlated with The Breast Cancer Progress: A Novel Biomarker for Differential Identification of Breast Cancer Tissues
Source: Cell J. 2021 Aug 29;23(4):414–20. doi: 10.22074/cellj.2021.7283 (PMC8405088; doi:10.22074/cellj.2021.7283)
Supplement: Supplementary file 1 [file Cell-J-23-414-s01.pdf]

CELL JOURNAL (Yakhteh)

Supplementary Information for

Downregulation of LINC02615 Is Correlated with The Breast Cancer Progress: A Novel Biomarker for Differential Identification of Breast Cancer Tissues

Sayed Rasoul Zaker, D.V.M.[1]*, Kamran Ghaedi, Ph.D.[2], [3]

1. Department of Plant and Animal Biology, Faculty of Biological Science and Technology, University of Isfahan, Isfahan, Iran
2. Department of Cellular Biotechnology, Cell Science Research Center, Royan Institute for Biotechnology, ACECR, Isfahan, Iran
3. Department of Cell and Molecular Biology and Microbiology, Faculty of Biological Science and Technology, University of Isfahan, Iran

**Corresponding Address: Department of Plant and Animal Biology, Faculty of Biological Science and Technology, University of Isfahan, Isfahan, Iran*
Email: r.zaker@sci.ui.ac.ir

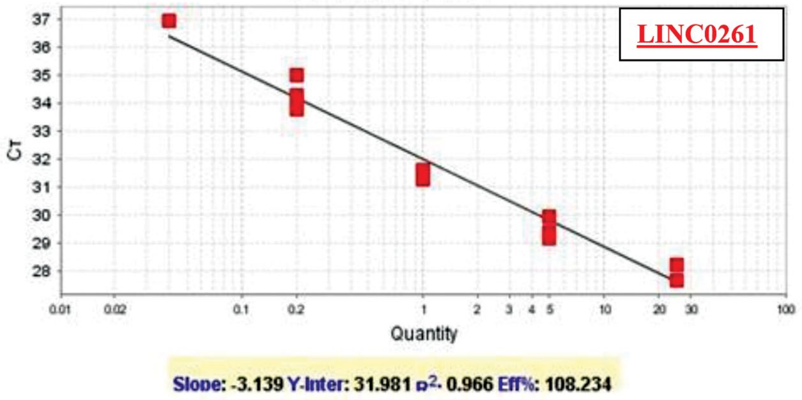

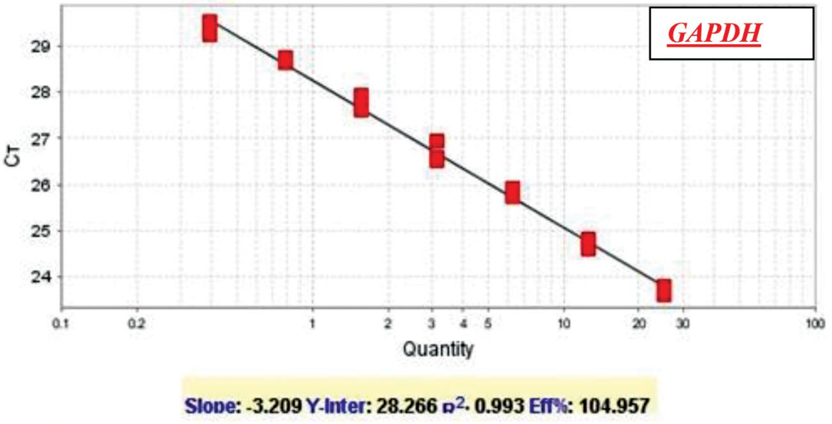

Fig.S1: Standard curve of the primer efficiencies used for quantitative reverse transcription polymerase chain reaction (qRT-PCR).

Table S1: Key genes explored in three important signaling pathways related to the pathogenesis of breast cancer and their roles in cancer

Gene names	Roles in cancer	Pathways
BRCA2	Tumor suppressor gene	Chromosomal instability
CHEK2	Tumor suppressor gene	Chromosomal instability
PALB2	Tumor suppressor gene	Chromosomal instability
BRIP1	Tumor suppressor gene	Chromosomal instability
RAD51	Tumor suppressor gene	Chromosomal instability
BRMS1	Tumor suppressor gene	Chromosomal instability
PTEN	Tumor suppressor gene	Proliferation survival translation
CASP8	Tumor suppressor gene	Apoptosis
BCAR1	Tumor suppressor gene	Apoptosis
ESR2	Tumor suppressor gene	Cell cycle progression
ESR1	Oncogene	Cell cycle progression
CCND1	Oncogene	Cell cycle progression
PIK3CA	Oncogene	Proliferation survival translation
AKT1	Oncogene	Proliferation survival translation
K-RAS	Oncogene	Proliferation survival translation
Braf	Oncogene	Proliferation survival translation

Table S2: The predicted miRNAs targeting the one-third of the selected key genes at the mRNA levels

miR names	Target genes
hsa-miR-19a-3p	*AKT1, CCND1, CHEK2, ESR1, PIK3CA, PTEN*
hsa-miR-155-5p	*AKT1, CCND1, KRAS, PIK3CA, PTEN, RAD51*
hsa-miR-193b-3p	*AKT1, CCND1, ESR1, KRAS, PTEN, RAD51*
hsa-miR-17-5p	*AKT1, BCAR1, BRCA2, BRIP1, CASP8, CCND1, ESR1, PIK3CA, PTEN*
hsa-miR-193b-3p	*AKT1, CCND1, ESR1, KRAS, PTEN, RAD51*
hsa-miR-3163	*BRIP1, CCND1, ESR1, KRAS, PIK3CA, PTEN, Braf*
hsa-miR-548c-3p	*BRIP1, CASP8, CCND1, KRAS, PIK3CA, PTEN, Braf*
hsa-miR-548e-5p	*BRIP1, CASP8, CHEK2, ESR1, PALB2, PIK3CA*
hsa-miR-590-3p	*BRIP1, CASP8, ESR1, KRAS, PIK3CA, PTEN*
hsa-miR-190a-3p	*BRIP1, CASP8, CCND1, KRAS, PIK3CA, PTEN*
hsa-miR-548x-3p hsa-miR-548aj-3p	*AKT1, BCAR1, CCND1, ESR1, KRAS, PIK3CA*
hsa-miR-19a-3p hsa-miR-19b-3p	*CASP8, CCND1, ESR1, KRAS, PIK3CA, PTEN*
hsa-miR-3148	*BCAR1, CCND1, KRAS, PIK3CA, PTEN, Braf*
hsa-miR-590-3pÂ	*BRCA2, BRIP1, CASP8, ESR1, KRAS, PIK3CA, PTEN, Braf*
hsa-miR-19aÂ hsa-miR-19bÂ	*BRCA2, CASP8, CCND1, ESR1, KRAS, PIK3CA, PTEN*
hsa-miR-548x-3p hsa-miR-548aj-3p	*AKT1, BCAR1, BRIP1, CASP8, CCND1, CHEK2, ESR1, KRAS, PIK3CA, PTEN, Braf*
hsa-miR-548ac	*AKT1, BRIP1, CASP8, CCND1, CHEK2, ESR1, ESR2, KRAS, PIK3CA, PTEN, Braf*

Table S2: Continued

miR names	Target genes
hsa-miR-548c-3p	*BRIP1, CASP8, CCND1, CHEK2, ESR1, KRAS, PALB2, PIK3CA, PTEN, Braf*
hsa-miR-548h-3p hsa-miR-548z	*AKT1, BRIP1, CCND1, CHEK2, ESR1, ESR2, KRAS, PIK3CA, PTEN, Braf*
hsa-miR-548as-3p	*AKT1, BCAR1, BRIP1, CCND1, ESR2, KRAS, PALB2, PIK3CA, PTEN, Braf*
hsa-miR-3613-3p	*BRIP1, CCND1, CHEK2, ESR1, KRAS, PALB2, PIK3CA, PTEN, Braf*
hsa-miR-548ae-3p hsa-miR-548ah-3p hsa-miR-548aq-3p hsa-miR-548j-3p hsa-miR-548am-3p	*AKT1, BRIP1, CCND1, CHEK2, ESR1, KRAS, PIK3CA, PTEN, Braf*
hsa-miR-548d-3p hsa-miR-548bb-3p	*AKT1, BRIP1, CCND1, ESR1, ESR2, KRAS, PIK3CA, PTEN, Braf*
hsa-miR-664b-3p hsa-miR-579-3p	*BRCA2, BRIP1, ESR1, KRAS, PALB2, PIK3CA, PTEN, Braf*
hsa-miR-153-5p	*BRCA2, BRIP1, ESR2, KRAS, PALB2, PIK3CA, PTEN, Braf*
hsa-miR-7856-5p	*BRCA2, ESR1, ESR2, KRAS, PALB2, PIK3CA, PTEN, Braf*
hsa-miR-4307	*BRCA2, CASP8, CCND1, ESR2, KRAS, PALB2, PIK3CA, PTEN*
hsa-miR-369-3p	*AKT1, BRCA2, BRIP1, CCND1, KRAS, PIK3CA, PTEN, Braf*
hsa-miR-3606-3p hsa-miR-590-3p	*BRCA2, BRIP1, CASP8, ESR1, KRAS, PIK3CA, PTEN, Braf*
hsa-miR-4698	*BRIP1, CASP8, CCND1, CHEK2, ESR1, KRAS, PIK3CA, PTEN*
hsa-miR-548e-5p	*AKT1, BRIP1, CASP8, ESR1, KRAS, PALB2, PIK3CA, Braf*
hsa-miR-548t-3p hsa-miR-548aa	*AKT1, BRIP1, CCND1, ESR2, KRAS, PIK3CA, PTEN, Braf*
hsa-miR-495-3p	*BRIP1, CASP8, CCND1, ESR1, KRAS, PIK3CA, PTEN, Braf*
hsa-miR-7853-5p hsa-miR-105-5p	*BCAR1, BRIP1, CASP8, ESR1, KRAS, PIK3CA, PTEN, Braf*
hsa-miR-335-3p	*BRCA2, CHEK2, KRAS, PALB2, PIK3CA, PTEN, Braf*
hsa-miR-5692a hsa-miR-3658	*BRCA2, BRIP1, KRAS, PALB2, PIK3CA, PTEN, Braf*
hsa-miR-548x-5p hsa-miR-548aj-5p hsa-miR-	*BRCA2, CASP8, ESR1, KRAS, PALB2, PIK3CA, PTEN*
hsa-miR-129-5p	*BRCA2, CCND1, KRAS, PALB2, PIK3CA, PTEN, Braf*
hsa-miR-3148	*BCAR1, BRCA2, CCND1, KRAS, PIK3CA, PTEN, Braf*
hsa-miR-1305	*CHEK2, ESR1, KRAS, PALB2, PIK3CA, PTEN, Braf*
hsa-miR-8063	*BRIP1, CASP8, CHEK2, KRAS, PIK3CA, PTEN, Braf*
hsa-miR-1277-5p	*BRIP1, CCND1, KRAS, PALB2, PIK3CA, PTEN, Braf*
hsa-miR-548f-3p hsa-miR-374a-5p	*AKT1, BRIP1, CCND1, KRAS, PIK3CA, PTEN, Braf*
hsa-miR-548ap-3p	*AKT1, BRIP1, CCND1, ESR2, PIK3CA, PTEN, Braf*
hsa-miR-4775	*BRIP1, ESR1, ESR2, KRAS, PIK3CA, PTEN, Braf*
hsa-miR-4753-3p	*BRCA2, BRIP1, CCND1, CHEK2, ESR2, Braf*
hsa-miR-4729	*BRCA2, BRIP1, ESR1, KRAS, PIK3CA, PTEN*

Table S2: Continued

miR names	Target genes
hsa-miR-513a-3p hsa-miR-4282 hsa-miR-944 hsa-miR-513c-3p	*BRCA2, BRIP1, KRAS, PIK3CA, PTEN, Braf*
hsa-miR-208b-5p	*CHEK2, KRAS, PALB2, PIK3CA, PTEN, Braf*
hsa-miR-186-5p	*BRIP1, CHEK2, ESR1, ESR2, PIK3CA, PTEN*
hsa-miR-1323	*BRIP1, CHEK2, KRAS, PIK3CA, PTEN, Braf*
hsa-miR-4482-3p	*BRIP1, CCND1, CHEK2, PIK3CA, PTEN, Braf*
hsa-miR-374b-5p	*BRIP1, CCND1, CHEK2, KRAS, PTEN, Braf*
hsa-miR-4694-3p	*BCAR1, BRIP1, KRAS, PALB2, PIK3CA, PTEN*
hsa-miR-548f-5p	*CASP8, ESR1, KRAS, PALB2, PIK3CA, PTEN*
hsa-miR-548az-5p hsa-miR-548t-5p	*ESR1, KRAS, PALB2, PIK3CA, PTEN, Braf*
hsa-miR-6835-3p	*CASP8 ESR1 KRAS PALB2 PIK3CA Braf*
hsa-miR-5590-3p	*CCND1 KRAS PALB2 PIK3CA PTEN Braf*
hsa-miR-142-5p	*CASP8, KRAS, PALB2, PIK3CA, PTEN, Braf*
hsa-miR-5692c hsa-miR-5692b hsa-miR-548e-3p hsa-miR-548az-3p	*BRIP1, CCND1, KRAS, PIK3CA, PTEN, Braf*
hsa-miR-494-3p	*CCND1, ESR2, PIK3CA, PTEN, RAD51, Braf*
hsa-miR-548aw	*CASP8, CCND1, ESR1, KRAS, PIK3CA, PTEN*
hsa-miR-651-3p	*CCND1, ESR1, KRAS, PIK3CA, PTEN, Braf*
hsa-miR-5582-3p	*BCAR1, CCND1, KRAS, PIK3CA, PTEN, Braf*
hsa-miR-106a-5p	CASP8, CCND1, ESR1, KRAS, PTEN, Braf

Table S3: Predicted miRNAs targeting most of the selected genes at mRNA levels

miR names	Target genes
hsa-miR-19a-3p	*AKT1, CCND1, CHEK2, ESR1, PIK3CA, PTEN, CASP8, KRAS, BRCA2*
hsa-miR-193b-3p	*AKT1, CCND1, ESR1, KRAS, PTEN, RAD51*
hsa-miR-548c-3p	*BRIP1, CASP8, CCND1, KRAS, PIK3CA, PTEN, Braf, ESR1, PALB2, CHEK2*
hsa-miR-548e-5p	*BRIP1, CASP8, CHEK2, ESR1, PALB2, PIK3CA, AKT1, Braf, KRAS*
hsa-miR-590-3p	*BRIP1, CASP8, ESR1, KRAS, PIK3CA, PTEN, Braf, BRCA2*
hsa-miR-17-5p	*AKT1, BRIP1, CASP8, CCND1, ESR1, PIK3CA, PTEN, BRCA2, BCAR1*
hsa-miR-548x-3p and hsa-miR-548aj-3p	*AKT1, BRIP1, CASP8, CCND1, ESR1, PIK3CA, BCAR1, CHEK2, KRAS, Braf*
hsa-miR-548ac	*AKT1, BRIP1, CASP8, CCND1, CHEK2, ESR1, ESR2, KRAS, PIK3CA, PTEN, Braf*

Table S4: The most important function of predicted miRNAs in cancer

miR names	-Log P value	P value	Function	GO ID
hsa-miR-17-5p; hsa-miR-193b-3p; hsa-miR-186-5p	1.306459	0.049379	Tumor necrosis factor receptor binding	GO0005164
hsa-miR-155-5p; hsa-miR-193b-3p; hsa-miR-19b-3p; hsa-miR-374a-5p; hsa-miR-186-5p	1.408032	0.039081	DNA damage response signal transduction resulting in induction of apoptosis	GO0008630
hsa-miR-155-5p; hsa-miR-17-5p; hsa-miR-193b-3p; hsa-miR-19a-3p; hsa-miR-19b-3p; hsa-miR-129-5p; hsa-miR-374a-5p; hsa-miR-186-5p; hsa-miR-374b-5p; hsa-miR-494-3p; hsa-miR-106a-5p	1.656198	0.02207	Positive regulation of apoptotic process	GO0043065
hsa-miR-155-5p; hsa-miR-17-5p; hsa-miR-193b-3p; hsa-miR-19a-3p; hsa-miR-19b-3p; hsa-miR-129-5p; hsa-miR-374a-5p; hsa-miR-513a-3p; hsa-miR-186-5p; hsa-miR-494-3p; hsa-miR-106a-5p	1.656198	0.02207	Negative regulation of apoptotic process	GO0043066
hsa-miR-155-5p; hsa-miR-17-5p; hsa-miR-193b-3p; hsa-miR-129-5p; hsa-miR-186-5p	1.705573	0.019698	Negative regulation of cell cycle	GO0045786
hsa-miR-155-5p; hsa-miR-17-5p; hsa-miR-193b-3p; hsa-miR-129-5p; hsa-miR-186-5p	1.705573	0.019698	Regulation of cell motility	GO2000145
hsa-miR-19b-3p; hsa-miR-590-3p	1.857332	0.013889	Regulation of tumor necrosis factor mediated signaling pathway	GO0010803
hsa-miR-155-5p; hsa-miR-17-5p; hsa-miR-193b-3p; hsa-miR-19a-3p; hsa-miR-19b-3p; hsa-miR-105-5p; hsa-miR-129-5p; hsa-miR-186-5p; hsa-miR-494-3p; hsa-miR-106a-5p	1.868908	0.013524	Induction of apoptosis	GO0006917
hsa-miR-155-5p; hsa-miR-17-5p; hsa-miR-193b-3p; hsa-miR-19a-3p; hsa-miR-19b-3p; hsa-miR-590-3p; hsa-miR-129-5p; hsa-miR-374a-5p; hsa-miR-186-5p; hsa-miR-374b-5p; hsa-miR-494-3p; hsa-miR-106a-5p	1.903865	0.012478	Apoptotic process	GO0006915
hsa-miR-155-5p; hsa-miR-17-5p; hsa-miR-193b-3p; hsa-miR-19a-3p; hsa-miR-19b-3p; hsa-miR-548d-3p; hsa-miR-590-3p; hsa-miR-374a-5p; hsa-miR-186-5p; hsa-miR-374b-5p; hsa-miR-494-3p; hsa-miR-106a-5p	1.903865	0.012478	Cell proliferation	GO0008283
hsa-miR-155-5p; hsa-miR-17-5p; hsa-miR-193b-3p; hsa-miR-19b-3p; hsa-miR-590-3p; hsa-miR-186-5p; hsa-miR-374b-5p; hsa-miR-106a-5p	2.172188	0.006727	Regulation of apoptotic process	GO0042981
hsa-miR-155-5p; hsa-miR-17-5p; hsa-miR-193b-3p; hsa-miR-19b-3p; hsa-miR-590-3p; hsa-miR-374a-5p; hsa-miR-186-5p; hsa-miR-106a-5p	2.172188	0.006727	Cell cycle checkpoint	GO0000075
hsa-miR-155-5p; hsa-miR-17-5p; hsa-miR-193b-3p; hsa-miR-19a-3p; hsa-miR-19b-3p; hsa-miR-590-3p; hsa-miR-129-5p; hsa-miR-186-5p; hsa-miR-374b-5p; hsa-miR-494-3p; hsa-miR-106a-5p	2.18038	0.006601	Positive regulation of cell Proliferation	GO0008284
hsa-miR-155-5p; hsa-miR-17-5p; hsa-miR-193b-3p; hsa-miR-19a-3p; hsa-miR-19b-3p; hsa-miR-590-3p; hsa-miR-129-5p; hsa-miR-186-5p; hsa-miR-374b-5p; hsa-miR-494-3p; hsa-miR-106a-5p	2.18038	0.006601	Negative regulation of cell proliferation	GO0008285
hsa-miR-17-5p; hsa-miR-193b-3p; hsa-miR-19a-3p; hsa-miR-19b-3p; hsa-miR-186-5p; hsa-miR-494-3p	2.181981	0.006577	Positive regulation of intrinsic apoptotic signaling pathway	GO2001244
hsa-miR-155-5p; hsa-miR-17-5p; hsa-miR-19a-3p; hsa-miR-19b-3p; hsa-miR-105-5p; hsa-miR-186-5p	2.181981	0.006577	Positive regulation of tumor necrosis factor biosynthetic process	GO0042535
hsa-miR-155-5p; hsa-miR-193b-3p; hsa-miR-19a-3p; hsa-miR-19b-3p; hsa-miR-105-5p; hsa-miR-186-5p	2.181981	0.006577	Positive regulation of tumor necrosis factor production	GO0032760
hsa-miR-155-5p; hsa-miR-17-5p; hsa-miR-193b-3p; hsa-miR-186-5p	2.271061	0.005357	Positive regulation of cell cycle arrest	GO0071158
hsa-miR-155-5p; hsa-miR-17-5p; hsa-miR-193b-3p; hsa-miR-19a-3p; hsa-miR-19b-3p; hsa-miR-186-5p; hsa-miR-494-3p; hsa-miR-106a-5p	2.526716	0.002974	Induction of apoptosis by extracellular signals	GO0008624

Table S4: Continued

miR names	-Log P value	P value	Function	GO ID
hsa-miR-155-5p; hsa-miR-17-5p; hsa-miR-193b-3p; hsa-miR-19a-3p; hsa-miR-19b-3p; hsa-miR-186-5p; hsa-miR-374b-5p; hsa-miR-106a-5p	2.526716	0.002974	Intrinsic apoptotic signaling pathway	GO0097193
hsa-miR-155-5p; hsa-miR-17-5p; hsa-miR-193b-3p; hsa-miR-19a-3p; hsa-miR-19b-3p; hsa-miR-590-3p; hsa-miR-129-5p; hsa-miR-186-5p; hsa-miR-106a-5p	2.889831	0.001289	Regulation of cell proliferation	GO0042127
hsa-miR-155-5p; hsa-miR-17-5p	3.033424	0.000926	Cellular response to tumor necrosis factor	GO0071356
hsa-miR-155-5p; hsa-miR-17-5p	3.033424	0.000926	Response to tumor necrosis factor	GO0034612
hsa-miR-155-5p; hsa-miR-17-5p; hsa-miR-193b-3p; hsa-miR-19a-3p; hsa-miR-374b-5p	3.093707	0.000806	Mammary gland epithelial cell proliferation	GO0033598
hsa-miR-155-5p; hsa-miR-17-5p; hsa-miR-193b-3p; hsa-miR-19a-3p; hsa-miR-374b-5p	3.093707	0.000806	Positive regulation of mammary gland epithelial cell proliferation	GO0033601
hsa-miR-155-5p; hsa-miR-17-5p; hsa-miR-193b-3p; hsa-miR-19a-3p; hsa-miR-19b-3p; hsa-miR-590-3p; hsa-miR-186-5p; hsa-miR-494-3p	3.49957	0.000317	Positive regulation of cell cycle	GO0045787
hsa-miR-155-5p; hsa-miR-193b-3p	3.510545	0.000309	Positive regulation of cell cycle cytokinesis	GO0071777
hsa-miR-155-5p; hsa-miR-193b-3p	3.510545	0.000309	Negative regulation of mammary gland epithelial cell proliferation	GO0033600
hsa-miR-155-5p; hsa-miR-17-5p; hsa-miR-193b-3p	4.93105	1.17E-05	Positive regulation of extrinsic apoptotic signaling pathway	GO2001238

Table S5: Co-expression of the selected protein-coding genes and LINC02615

Gene names	Co-expression	Co-efficiency
BRCA2	Negative	-0.1943
CHEK2	Negative	-0.2563
PALB2	Positive	0.0931
BRIP1	Negative	-0.2456
RAD51	Negative	-0.3322
BRMS1	Negative	-0.2105
PTEN	Positive	0.1372
CASP8	Positive	0.1002
BCAR1	Negative	-0.1854
ESR2	Positive	0.1883
ESR1	Positive	0.1907
CCND1	Positive	0.1064
PIK3CA	Positive	0.1178
AKT1	Negative	-0.1354
K-RAS	Negative	-0.1659
Braf	Positive	0.1309

Table S6: The expression profile of LINC02615 in breast cancer patients and healthy individuals

Variable	n=24	LINC02615 Downregulation (n)	LINC02615 Upregulation (n)	P value	Cutoff point
Groups				0.043[a]	0.396
Healthy control	24	8	16		
BC patients	24	15	9		
HER2 expression				0.439	0.195
Positive	4	1	3		
Negative	2	0	2		
ER expression				0.014[a]	0.222
Positive	3	0	3		
Negative	3	3	0		
PR expression				0.083	0.222
Positive	2	0	2		
Negative	4	3	1		
Tumor stage				0.078	0.195
I/II	8	0	8		
III/IV	6	2	4		
Tumor grade				0.08	0.222
I/II	12	2	10		
III	3	2	1		
Tumor size (cm)				0.252	0.222
≤5	12	6	6		
≥5	15	1	4		
Family history of BC				0.273	0.222
Positive	3	0	3		
Negative	3	1	2		
Physical activity				0.028[a]	0.222
High	4	0	4		
Intermediate/Low	4	3	1		
Obesity				0.047[a]	0.195
Yes	11	1	10		
No	13	6	7		
Dietary factors					
1- Fat intake				0.621	7.571
High	6	4	2		
Intermediate/Low	5	4	1		

Table S6: Continued

Variable	n=24	LINC02615 Downregulation (n)	Upregulation (n)	P value	Cutoff point
High/Intermediate	5	3	2		
Low	6	5	1		
3- Dairy intake				0.121	0.396
High/Intermediate	10	4	6		
Low	2	2	0		
Income				0.209	0.396
High	2	0	2		
Intermediate	4	3	1		
Low	5				
Diabetes disease				0.046[a]	0.396
Yes	2	0	2		
No	2	2	0		
Stress				0.047[a]	7.571
Yes	8	7	1		
No	1	0	1		
Marital status				-	-
Married	16	9	7		
Never married/single	0	0	0		
Marital age				0.473	0.195
≤20	13	2	11		
≥20	3	1	2		
Age at first pregnancy (Y)				0.448	0.195
≤20	12	2	10		
≥20	3	0	3		
Breastfeeding history				0.067	0.396
Yes	10	7	3		
No	2	0	2		
Birth control pills (hormonal)				0.338	0.195
Yes	8	2	6		
No	3	0	3		
Age at menarche (Y)				-	-
≤15	14	8	6		
≥15	0	0	0		
Age at menopause (Y)				0.025[a]	7.571
≤47	2	2	0		
≥47	3	0	3		

[a]; P<0.05, BC; Breast cancer, ER; Estrogen receptor, PR; Progesterone receptor, and HER2; Human epithelial growth factor receptor 2.
